# Deep Investigating the Changes of Gut Microbiome and Its Correlation With the Shifts of Host Serum Metabolome Around Parturition in Sows

**DOI:** 10.3389/fmicb.2021.729039

**Published:** 2021-09-17

**Authors:** Hao Fu, Maozhang He, Jinyuan Wu, Yunyan Zhou, Shanlin Ke, Zhe Chen, Qin Liu, Min Liu, Hui Jiang, Lusheng Huang, Congying Chen

**Affiliations:** State Key Laboratory of Pig Genetic Improvement and Production Technology, Jiangxi Agricultural University, Nanchang, China

**Keywords:** gut microbiome, parturition, metagenomics, metabolomics, sow

## Abstract

Parturition is a crucial event in the sow reproduction cycle, which accompanies by a series of physiological changes, including sex hormones, metabolism, and immunity. More and more studies have indicated the changes of the gut microbiota from pregnancy to parturition. However, what bacterial species and functional capacities of the gut microbiome are changed around parturition has been largely unknown, and the correlations between the changes of gut bacterial species and host metabolome were also uncovered. In this study, by combining 16S rRNA gene and shotgun metagenomic sequencing data, and the profiles of serum metabolome and fecal short-chain fatty acids (SCFAs), we investigated the changes of gut microbiome, serum metabolite features and fecal SCFAs from late pregnancy (LP) to postpartum (PO) stage. We found the significant changes of gut microbiota from LP to PO stage in both 16S rRNA gene sequencing and metagenomic sequencing analyses. The bacterial species from *Lactobacillus*, *Streptococcus*, and *Clostridium* were enriched at the LP stage, while the species from *Bacteroides, Escherichia*, and *Campylobacter* had higher abundances at the PO stage. Functional capacities of the gut microbiome were also significantly changed and associated with the shifts of gut bacteria. Untargeted metabolomic analyses revealed that the metabolite features related to taurine and hypotaurine metabolism, and arginine biosynthesis and metabolism were enriched at the LP stage, and positively associated with those bacterial species enriched at the LP stage, while the metabolite features associated with vitamin B6 and glycerophospholipid metabolism had higher abundances at the PO stage and were positively correlated with the bacteria enriched at the PO stage. Six kinds of SCFAs were measured in feces samples and showed higher concentrations at the LP stage. These results suggested that the changes of gut microbiome from LP to PO stage lead to the shifts of host lipid, amino acids and vitamin metabolism and SCFA production. The results from this study provided new insights for the changes of sow gut microbiome and host metabolism around parturition, and gave new knowledge for guiding the feeding and maternal care of sows from late pregnancy to lactation in the pig industry.

## Introduction

Pregnancy and parturition are two extremely important reproduction stages to sows, which accompany the changes of hormone levels, metabolism, immunity, and even gut microbiome (Newbern and Freemark, 2011; Koren et al., 2012). As we have known, parturition is initiated by multiple, parallel, and interactive physiological factors that lead to uterine contractions and cervical remodeling following the dramatic changes of sex hormones. Mammals maintain a high level of serum progesterone concentration throughout the gestation period, and the decreased progesterone level is linked to the onset of labor (O’Brien, 1995; Word, 2007). Furthermore, each stage of the pregnancy and parturition is faced with an array of immunological challenges (Mor and Cardenas, 2010). Pro-inflammatory cytokines, particularly, the components of NF-κB signaling pathway, are expressed at the fetal-maternal interface during human pregnancy and labor (Lappas et al., 2002; Lappas and Rice, 2004). Many stimuli including maternal signals, intrauterine hypoxia, and intrauterine bacterial infection usually induce the activation of resident macrophages or lymphocytes within intrauterine tissues and the secretion of pro-inflammatory cytokines (e.g., IL-1β, IL-6, and TNF-α) or bacterial byproducts (e.g., LPS) in the amniotic fluid during labor (Romero et al., 1992; Cox et al., 1993; Baggia et al., 1996).

All these reports above have suggested the changes of gut microbiota during pregnancy and parturition. Previous studies have indicated that maternal inflammation and insulin sensitivity can alter the composition of gut microbiome during pregnancy (Vijay-Kumar et al., 2010; Koren et al., 2012). Accumulated evidences have shown that the changes in hormones and immunity can lead to the changes of gut microbiota which conversely affect the production of hormones and inflammatory factors (Lappas and Rice, 2007; Newbern and Freemark, 2011). Moreover, Koren et al. (2012) suggested that the gut microbiome undergoes a significant shift at the third trimester of pregnancy. Huang et al. (2019) further revealed a dramatic remodeling of the gut microbiota from late pregnancy to the postpartum stage by 16S rRNA gene sequencing. However, all these studies have revealed the changes of gut microbiota around parturition only at the operational taxonomic unit (OTU) level. To our knowledge, there are few studies investigating the changes in microbial composition and functional capacity of the gut microbiome around parturition with the metagenomic sequencing method.

In addition, many studies have indicated that certain microbial metabolites, including short-chain fatty acids (SCFAs), secondary bile acids and lipopolysaccharides can predict the changes in gut microbial composition, regulate immune functions, affect the body’s physiological state, and cause a wide range of diseases (Nicolas and Chang, 2019; Fan and Pedersen, 2020). What’s more, the metabolites produced by maternal gut microbiota during pregnancy play important roles in immune system development, energy metabolism, and susceptibility to inflammatory disease of the fetus (Nabhani and Eberl, 2020). For example, SCFAs in the colonic lumen of pregnant mice can shape the energy metabolism of embryos, and maintain postnatal energy homeostasis by the SCFA-GPR41 and SCFA-GPR43 axes (Kimura et al., 2020). However, the cross-talk between maternal gut microbiota and serum metabolites around peripartum remains undefined.

In this study, to systematically elucidate the changes of gut microbiota and its potential function capacity from the stage of late pregnancy (∼5 days before parturition, defined as the LP stage) to the stage of postpartum (within 24 h after delivery, defined as the PO stage), we performed both 16S rRNA gene and shotgun metagenomic sequencing of fecal samples from 96 sows. Meanwhile, to evaluate the shifts of serum and fecal metabolites around the parturition, and test the correlations between the changes of the gut microbiome and the shifts of metabolomic profiles, serum metabolomic profiles and fecal SCFAs were measured in experimental sows between LP and PO stage.

## Materials and Methods

### Experimental Animals and Sample Collection

A total of 96 sows from the intercross of Berkshire × Chinese indigenous pig breeds (Licha, Yushan, and Luchuan) were used in this study. Among them, 66 sows were from Berkshire × Licha F_1_ population. All experimental sows were raised in the same farm. Feces samples were obtained from these 96 sows at two time points between 5 days before parturition and within 24 h after delivery in autumn, 2017 (within 1 month from September to October). The information of parities and the exact sampling times are shown in Supplementary Table 1. Eventually, a total of 156 fecal samples were collected. Among 96 sows, 60 sows were obtained fecal samples at both LP and PO stages, 6 sows only had fecal samples at the LP stage, and 30 sows were only obtained fecal samples at the PO stage. All experimental sows were raised in independent pens separately and provided the same formula feed containing 17.5% crude protein, 0.91% lysine, and 3,300 kJ digestible energy twice daily. No feed changes occurred between two times of sampling. All sows showed normal feed intake. Water was available *ad libitum* from nipple drinkers. Experimental sows were healthy and did not receive probiotic or antibiotic therapy within 2 months of sample collection. Fecal samples were collected from the anus and immediately dipped in liquid nitrogen for transportation. And then, all samples were stored at −80∘C until use. A total of 115 blood samples were harvested from these 96 sows at the two-time points when feces samples were collected, including 28 samples at the LP stage and 87 samples at the PO stage. Serum samples were isolated by centrifugation (8,000 × *g*) for 10 min at 4∘C after coagulation and stored at −80∘C until use. All the information about experimental sows and sample collection is shown in Supplementary Table 1.

### Ethics Statement

All animal procedures were conducted according to the guidelines for the care and use of experimental animals that were established by the Ministry of Agriculture and Rural Affairs (MARA) of China. The approval for pig husbandry and experiment was obtained from Animal Care and Use Committee (ACUC) of Jiangxi Agricultural University (No. JXAU2011-006).

### Microbial DNA Extraction of Fecal Samples and 16S rRNA Gene Sequencing

Microbial DNA was extracted from fecal samples using QIAamp Fast DNA Stool Mini Kit (Qiagen, Germany) following the manual. The concentration and quality of DNA were measured by 0.8% agarose gel electrophoresis and the NanoDrop-1000 (Thermo Fisher Scientific, United States). The V3–V4 hypervariable region of the 16S rRNA gene was amplified with the barcode fusion primers (338F: 5-ACTCCTACGGGAGGCAGCAG-3, 806R:5-GGACTACHVGGGTWTCTAAT-3). After purification, PCR products were used for library construction and sequenced on an Illumina MiSeq platform (Illumina, United States). All 16S rRNA gene sequencing data was submitted to the China National GeneBank database with the accession number CNP0001534. The quality control of the 16S rRNA gene sequencing data was performed by filtering barcode, primer sequences and low-quality reads using QIIME (v1.9.1) pipeline (Kuczynski et al., 2011) as described previously (Huang et al., 2019). Pair-end clean sequences were then merged into tags using FLASH (v1.2.11) (Magoc and Salzberg, 2011). To avoid the influence of sequencing depth in statistical analysis, we rarefied the library size to 10,000 tags for all 156 samples. Tags were clustered into operational taxonomic unit (OTUs) at 97% of sequence similarity after chimeras were removed using USEARCH (v7.0.1090) (Edgar, 2010). Representative sequence of each OTU was then matched to the Greengenes database (v2013.5.99) (DeSantis et al., 2006) using the RDP classifier (v2.2) to obtain the microbial taxonomy information (Wang et al., 2007). The α-diversity indexes of gut microbiome including observed species, Chao, Ace, Simpson and Shannon were calculated by Mothur (v1.39.5) (Schloss et al., 2009).

### Metagenomic Sequencing and Bioinformatic Analysis

A total of 63 samples from 50 sows, which were included in 156 fecal samples described above, were used for metagenomic sequencing. There were 13 sows having fecal samples at both LP and PO stages. These 63 feces samples were comprised of 34 samples at the LP stage and 29 samples at the PO stage. The DNA libraries were constructed with an insert size of 350 base pairs (bp) for each sample and then sequenced with a PE 150-bp strategy on a Novaseq 6000 platform (Illumina, United States). All metagenomic sequencing data was submitted to the China National GeneBank database with the accession number CNP0000824. Raw reads were filtered out the adapter sequences and low-quality reads by fastp (v0.19.4) (Chen et al., 2018). Host genomic DNA sequences were removed from data through blasting to the swine reference genome sequence (Sscrofa11.2) by BWA (v0.7.17) software (Li and Durbin, 2009). The clean reads of each sample were assembled by MEGAHIT (v1.1.3) with the option “–min-count 2 –k-min 27 –k-max 87 –k-step 10–min-contig-len 500” (Li et al., 2016). The contigs with length ≥ 500 bp were used to predict open reading frames (ORFs) using MetaGeneMark (v3.38) software (Zhu et al., 2010). A non-redundant gene catalog was generated by clustering all predicted genes at 95% sequence identity of amino acids and 90% coverage with CD-HIT (v4.7) (Li and Godzik, 2006). Taxonomic assignments were processed by DIAMOND (v0.9.24) (Buchfink et al., 2014) against the NCBI-NR (version April 2019) database, and then classified by BASTA (v1.3.2.3) based on the last common ancestor algorithms. KEGG databases were used for the functional classification of metagenomic genes (Ogata et al., 1999). The abundance of function terms of KEGG pathways was determined by KOBAS (v2.0) software (Xie et al., 2011).

### Untargeted Metabolome Measurements of Sow Serum Samples

The serum samples were thawed on ice and prepared 100 μL for metabolome measurement. Three hundred microliters of pre-cooled methanol (Merk Corp., Germany) were added into 100 μL of serum sample, and then vortexed for 1 min. After incubated at −20∘C for 20 min, the mixtures were centrifuged at 15,000 × *g* (rcf) for 15 min at 4∘C. Supernatants were transferred into clean tubes and dried in a Savant vacuum evaporator (Thermo Fisher Scientific, United States). Dried supernatants were resolved in 150 μL of the mixed solvent of water and methanol (85:15, v/v), and then, placed into the sampling vials for the next measurement. At the same time, a quality control (QC) sample was prepared by combining aliquots of an equal volume of each tested sample. All tested samples and the QC sample were measured in a randomized order by an ultra-performance liquid chromatography coupled with quadrupole time-of-flight mass spectrometry (UPLC-QTOF/MS) (Waters Crop., United States).

Working solution (1.0 μL) was injected into a 100 mm × 2.1 mm, 1.7 μm BEH C18 column (Waters Corp., United States) and held at 40∘C under the UPLC system (Waters Crop., United States). The QC sample was injected eight times at the beginning of the run to ensure system equilibrium, and then injected every 12 tested samples to further monitor the stability of measurement. For both positive electrospray ion mode (ES^+^) and negative electrospray ion mode (ES^–^), serum samples were eluted using a linear gradient from 100% A to 100% B (A, water + 0.1% formic acid; B, acetonitrile) at a flow rate of 0.3 mL/min at the column temperature of 40∘C for 18 min in ES^–^ (22 min in ES^+^) as described previously (He et al., 2019a). Mass spectrometric data were collected using a Waters Q-TOF Premier (Waters Corp., United States) equipped with an electrospray source operating in either ES^+^ or ES^–^. The source temperature was set at 120∘C and the desolvation gas temperature was set at 350∘C. The capillary voltage was set at 3.0 kV for ES^+^ and 2.5 kV for ES^–^. Mass scanning ranged from 50 to 1,200 m/z with a scan time of 0.3 s and an interscan delay of 0.02 s over the scanning time.

MassLynx software (Waters Corp., United States) was used for data acquisition and system control. Leucine-enkephalin was used as an external standard at a concentration of 100 ng/mL and a flow rate of 5 μL/min in all analyses. Progenesis QI software (v2.0) (Non-linear Dynamics, United Kingdom) was used for peak selection and grouping, retention time correction, second peak grouping, and isotope and adducts annotation (Rusilowicz, 2016). We obtained a peak list containing the retention time, m/z, and peak area for each sample after peak alignment by Progenesis QI (Li et al., 2014). And then, we acquired ion intensities of each peak and generated a matrix containing arbitrarily assigned peak indices (retention time-m/z pairs), ion intensities (variables), and sample names (observations) (Liu et al., 2019). Raw matrices were further filtered by keeping those non-zero values of measurements that existed in more than 20% of the samples and 50% of the QC sample (Bijlsma, 2006). The molecular mass data (m/z) was aligned with the HMDB database to identify relevant metabolites (Wishart et al., 2018). Because all biological samples were collected and detected in the same batch, we kept those metabolites for which coefficient of variation (CV) was less than 30% in the QC samples for further analysis (Liu et al., 2019).

### Measurement of Fecal Short-Chain Fatty Acids by Gas Chromatograph

We measured the concentrations of SCFAs including acetic acid, propionic acid, isobutyric acid, butyric acid, isopentanoic acid, and pentanoic acid in all 156 fecal samples (66 samples at the LP stage and 89 samples at the PO stage) from 96 sows. In brief, 0.3 g of each lyophilized fecal sample was weighted and mixed with 1,500 mL of DNase/RNase-Free distilled water. And then, the mixture was homogenized for 30 s, followed by centrifugation at 5,000 rpm for 4 min. We pipetted 1,200 μL of supernatant into a new microtube of 1.5 mL. Two hundred and forty microliters of mixture of metaphosphoric acid and botenic acid (1:1, v/v) was added into the supernatant, vortexed and centrifugated at 15,000 rpm for 15 min. The supernatant was absorbed by a disposable syringe and filtered by a 0.22-μm filter membrane (Millipore Express, Germany). A total of 1,000 μL of filtrate was accurately transferred into a GC vial. Pure water was used as blank control to correct the background. Preprocessed samples and blank sample were loaded to a GC-2014 gas chromatograph (Shimadzu, Japan) equipped with a flame ionization detection and a thin-film capillary column DB-FFAP 30 m × 0.25 μm × 0.25 μm (Shimadzu, Japan). LabSolutions software (Shimadzu, Japan) was used for data processing.

## Statistical Analysis

### Comparison of the Phylogenetic Composition and Functional Capacity of Sow Gut Microbiome Between Late Pregnancy and Postpartum Stage

The β-diversity of gut microbiota was analyzed by QIIME (v1.9.1) (Kuczynski et al., 2011). Principle coordinate analysis (PCoA) was performed to compare the phylogenetic composition of sow gut microbiota between LP and PO stage based on Bray-Curtis distances by the vegan in R package (Dixon, 2003). The permutational multivariate analysis of variance (PERMANOVA) was used to test group differences of gut microbial composition. Wilcoxon rank sum test (unpaired) was used to identify the bacterial taxa and KEGG pathways showing differential abundances between LP and PO stage (MacFarland and Yates, 2016). Spearman’s rank correlation was applied to test the correlation between differential KEGG pathways and microbial taxa (Krumsiek et al., 2011).

### Construction of Modules of Serum Metabolite Features, and Identification of Differential Metabolic Features Between Late Pregnancy and Postpartum Stage

We obtained 15,965 metabolite features in the tested samples. After filtering at the threshold of coefficient of variation (CV) ≤ 30%, a total of 7,857 metabolite features were left for further analysis. These metabolite features were normalized by log_10_ transformation of the m/z values and used to construct the co-abundance topological network of serum metabolites by the weighted correlation network analysis (WGCNA) in R package with scale-free topology criterion soft threshold of β = 6 (Langfelder and Horvath, 2008). Metabolic modules were extracted from topological networks by the dynamic hybrid tree-cutting algorithm with the parameters of deepSplit = 4 and minModuleSize = 60 (Langfelder et al., 2008). Similar modules were merged if the Pearson correlation coefficient exceeded 0.75 between the eigen vectors of the serum metabolite clusters. We first analyzed the correlations between metabolite modules and differential bacterial species by Spearman’s rank correlation analysis. And then, we chose those metabolite features which were contained in the modules correlated with differential bacterial species, could be annotated into KEGG pathways by online MetaboAnalyst 4.0, and showed differential abundances between LP and PO stage at the threshold of FDR < 0.05 by Wilcoxon rank sum test. Finally, we tested the Spearman’s rank correlations between the metabolite features chosen and bacterial species showing differential abundances between LP and PO stage. Meanwhile, 1,911 metabolite features showing differential abundance between LP and PO stage were identified with a variable importance on the projection (VIP) score > 1 by sparse partial least squares-discriminant analysis (sPLS-DA) using the online tool MetaboAnalyst 4.0 (Chong et al., 2018). All differential metabolites were used for KEGG enrichment analysis with MetaboAnalyst (v4.0).

## Results

### Alteration of Sow Gut Microbiota From Late Pregnancy and Postpartum Stage With 16S rRNA Gene Sequencing Data

At the threshold of relative abundance > 0.05%, a total of 311 OTUs were used for further analyses. Most of experimental sows (90 sows) were at the parity 1–3 (Supplementary Table 1). We first analyzed the effect of parity on the gut microbial compositions. However, no significant effect was detected (Supplementary Figure 1). Because the actual delivery time was different from the predicted date of parturition, the intervals from the sampling time to delivery time were different. According to the intervals from feces sampling to delivery, we divided all feces samples into four groups: samples collected within 1∼5 days before parturition, samples collected within 24 h before parturition, samples collected within 12 h after parturition, and samples collected within 12∼24 h after parturition. And then, we analyzed the changes of the gut microbiota following the longitudinal time series described above. However, no significant shifts of the gut microbiota were identified between the samples collected within 24 h before parturition and within 1∼5 days before parturition, and between the samples harvested within 12 h and within 12∼24 h after parturition (Supplementary Figure 2). Therefore, we only focused on the comparison of gut microbiome between LP and PO stages in the following analyses.

**FIGURE 1:**
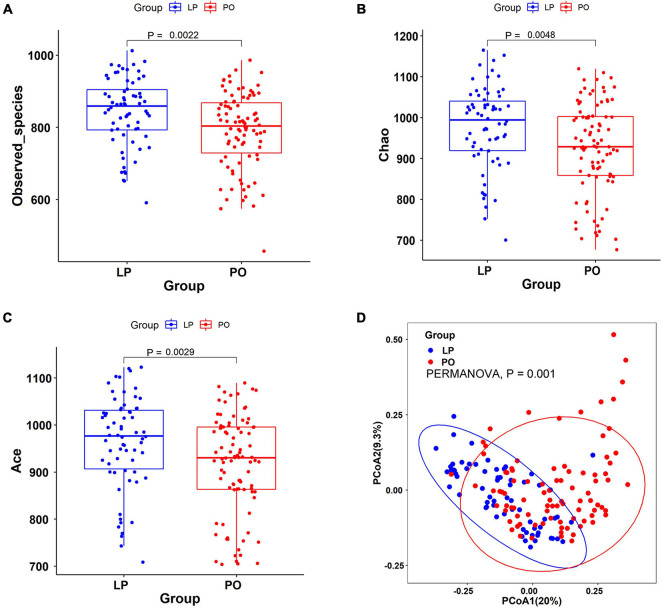
Significant changes of gut microbial community structures from late pregnancy (LP) to postpartum (PO) stage based on the α- and β-diversity with 16S rRNA gene sequencing data. **(A)** Observed species. **(B)** Chao index. **(C)** Ace index. **(D)** Principal coordinate analysis (PCoA) based on Bray-Curtis distances. PERMANOVA, the permutational multivariate analysis of variance.

We observed significantly higher α-diversity of sow gut microbiota at the LP stage compared to that at the PO stage, including Sobs, Ace and Chao index (Figures 1A–C). PCoA showed significant alteration of the gut microbial community structures from LP to PO stage based on the Bray-Curtis distances (PERMANOVA, *P* = 0.001) (Figure 1D). We identified a total of 12 phyla, 39 genera and 167 OTUs showing a drastic change in their relative abundance from LP to PO stage (Supplementary Figure 3 and Supplementary Table 2). For examples, at the phylum level, *Bacteroidetes*, and *Fibrobacteres* were enriched at the LP stage, while *Firmicutes* and *Fusobacteria* had higher abundance at the PO stage. At the genus level, *Prevotella*, *CF231*, and *Clostridium* were enriched at the LP stage, while *Bacteroides* and *Escherichia* were enriched at the PO stage. At the OTU level, 32 OTUs belonging to *Prevotella* were enriched at the LP stage, while 7 OTUs from *Bacteroides* and 22 OTUs annotating to *Ruminococcaceae* had higher abundance at the PO stage.

### Identification of Bacterial Species Showing Differential Abundances Between Late Pregnancy and Postpartum Stages

**FIGURE 2:**
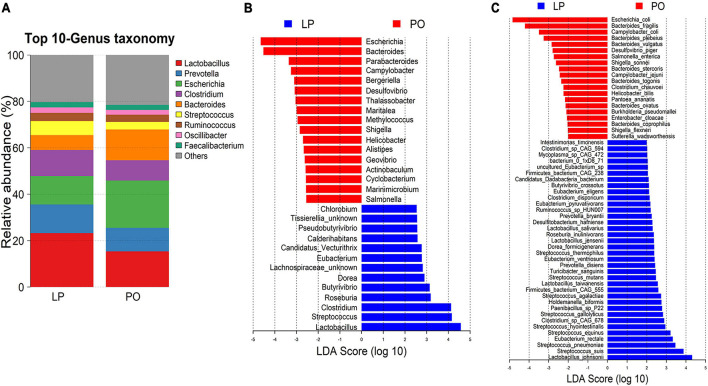
Gut microbial composition and differential bacteria between late pregnancy (LP, *n* = 34) and postpartum (PO, *n* = 29) stages based on shotgun metagenomic sequencing data. **(A)** Relative abundances of the top 10 bacterial genera at the LP and PO stage. **(B)** Differential bacterial genera between LP and PO stages by LEfSe analysis. LDA score > 2. **(C)** Differential bacterial species between LP and PO stages by LEfSe analysis. LDA Score > 2. LDA, linear discriminant analysis.

With metagenomic sequencing data, a total 366 bacterial species with relative abundance > 0.005% and present in at least 20% of all tested samples were used to identify the bacterial species having differential abundances between two stages. A total of 30 genera and 56 bacterial species showed distinct abundances between LP and PO stages (Figure 2 and Supplementary Table 3). At the genus level, 13 genera had a higher abundance at the LP stage, mainly including *Lactobacillus*, *Streptococcus*, and *Clostridium*. Conversely, 17 genera showed an increased abundance at the PO stage, including *Escherichia* and *Bacteroides* (Figures 2A,B). This was consistent with the result obtained from 16S rRNA gene sequencing data. At the species level, 36 species had significantly higher abundance at the LP stage, mainly including *Lactobacillus johnsonii*, *Streptococcus suis*, and *Eubacterium rectale*, while 20 species showed enrichments at the PO stage, mainly including *Escherichia coli*, *Bacteroides fragilis*, and *Campylobacter coli* (Figure 2C). Together, these results demonstrated that the composition and abundance of gut microbiota were significantly changed from LP to PO stage.

### The KEGG Pathways Showing Differential Enrichments Between Late Pregnancy and Postpartum Stages

**FIGURE 3:**
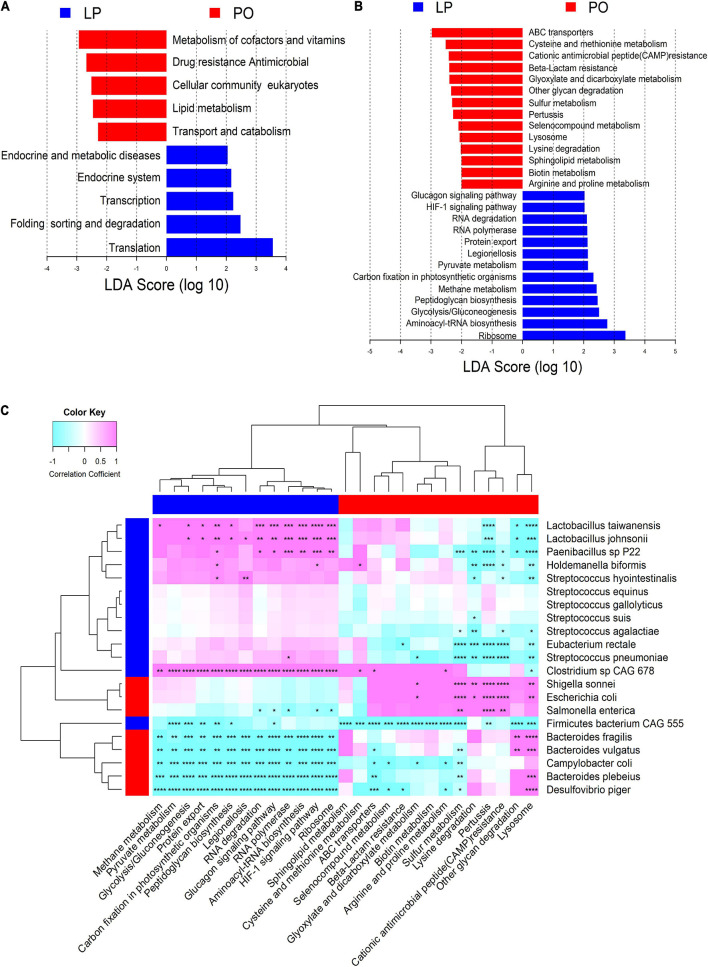
KEGG pathways enriched in each of late pregnancy (LP) and postpartum (PO) stage and their correlations with differential bacterial species. Differential KEGG pathways between LP and PO stages at the level 2 **(A)** and level 3 **(B)** by LEfSe analysis. LDA, linear discriminant analysis. **(C)** Heatmap showing the correlations between differential KEGG pathways of gut microbiota and differential bacterial species. The stars indicate the significant threshold *P < 0.05, **P < 0.01, ***P < 0.001, and ****P < 0.0005 by Spearman’s rank correlation test.

We identified a total of 10 Kyoto Encyclopedia of Genes and Genomes (KEGG) pathways at the level 2 and 27 KEGG pathways at the level 3 showing different enrichments between LP and PO stages (Figures 3A,B). The pathways related to bacterial cell division and reproduction (e.g., transcription and translation) were significantly enriched at the LP stage, whereas the pathways associated with metabolisms, such as the metabolisms of lipid, cofactors, and vitamins were predominantly enriched at the PO stage. Furthermore, we found that the pathways related to glucose metabolism, such as glycolysis/gluconeogenesis, pyruvate metabolism, HIF-1 signaling pathway, and glucagon signaling pathway were enriched at the LP stage. However, the pathways associated with drug resistance for antimicrobials, such as cationic antimicrobial peptide (CAMP) resistance and beta-lactam resistance had higher abundances at the PO stage, suggesting pathogens infection and antibiotic resistance occurring at the PO stage. We also investigated the correlations between bacterial species and KEGG functional pathways showing differential abundances between LP and PO stages by spearman’s correlation analysis (Figure 3C). *Lactobacillus johnsonii*, *Lactobacillus taiwanensis*, and *Paenibacillus* sp. *P22* which had higher abundances at the LP stage were positively correlated with the pathways about ribosome, aminoacyl-tRNA biosynthesis, RNA polymerase, RNA degradation, and peptidoglycan biosynthesis that were also enriched at the LP stage. Those bacteria enriched at the PO stage, including *Bacteroides plebeius*, *Bacteroides fragilis*, *Bacteroides vulgatus*, and *Desulfovibrio piger*, were negatively correlated to these pathways. Additionally, *Escherichia coli*, *Shigella sonnei*, and *Salmonella enterica* were positively correlated with the pathways of CAMP resistance, pertussis, lysosome, and sulfur metabolism. Interestingly, *Bacteroides fragilis* and *Bacteroides vulgatus* were positively associated with the pathways of other glycan degradation and lysosome (Figure 3C).

### Distinct Serum Metabolome and Fecal Short-Chain Fatty Acid Concentrations Between Late Pregnancy and Postpartum Stages

**FIGURE 4:**
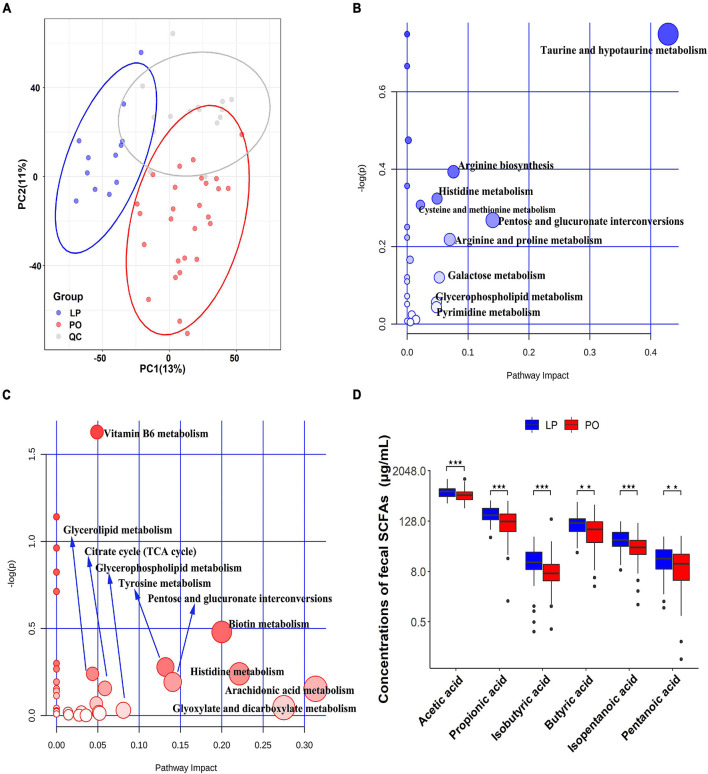
Distinct serum metabolome and concentrations of fecal SCFAs between late pregnancy (LP) and postpartum (PO) stages. **(A)** Principal component analysis (PCA) indicates the significant changes of host serum metabolome from LP to PO stage. QC, quality control sample. **(B)** The KEGG pathways enriched by metabolite features having higher abundance at the LP stage. **(C)** The KEGG pathways enriched by metabolite features having higher abundance at the PO stage. *Y*-axis indicates the *p*-values obtained from enrichment analysis. *X*-axis shows the impact values from the pathway topology analysis. The size and color of nodes indicate the value of pathway impact. **(D)** Comparison of the concentrations of fecal short-chain fatty acids (SCFAs) between LP and PO stages in 155 samples. Wilcoxon rank-sum test, **P < 0.01 and ***P < 0.001.

We measured metabolomic profiles of 39 serum samples from 35 sows for which metagenomic sequencing data were also available, including 12 samples at the LP stage and 27 samples at the PO stage. Among 35 sows, four sows had serum samples at both LP and PO stages. After quality control based on CV less than 30%, we identified 7,857 quantifiable serum metabolites (m/z) from positive ion mode (3,627) and negative ion mode (4,230) for further analysis. Parities did not either show significant effect on serum metabolome profiles (Supplementary Figure 4). We first performed a PCA analysis to evaluate the global shifts in serum metabolome from LP to PO stage after data transformation (log10) and auto-scaling. An obvious shift in metabolomic profiles was observed from LP to PO stage (Figure 4A). A total of 1,911 differential metabolites were identified at an optimal threshold of VIP > 1. Among them, 1,539 differential metabolites could be annotated by HMDB database. We revealed specific metabolomic profiles at each stage after we matched metabolite features to metabolic pathways by online MetaboAnalyst 4.0 (Figures 4B,C and Supplementary Table 4). The pathways of taurine and hypotaurine metabolism, and arginine biosynthesis and metabolism were significantly enriched at the LP stage. However, vitamin metabolism (e.g., vitamin B6 and biotin) and lipid metabolism (e.g., glycerolipid and glycerophospholipid) had higher abundance at the PO stage. We also found that the pathways of histidine metabolism, and pentose and glucuronate interconversions were enriched by differential metabolites at both LP and PO stages. Additionally, we measured the concentrations of SCFAs in 115 fecal samples including 66 samples at the LP stage and 89 samples at the PO stage. We found that the concentration of all six kinds of SCFAs was significantly higher at the LP stage (Wilcoxon rank-sum test, *P* < 0.01) (Figure 4D).

### Contribution of the Changes of Gut Microbiome to the Shifts of Serum Metabolome and Fecal Short-Chain Fatty Acids

**FIGURE 5:**
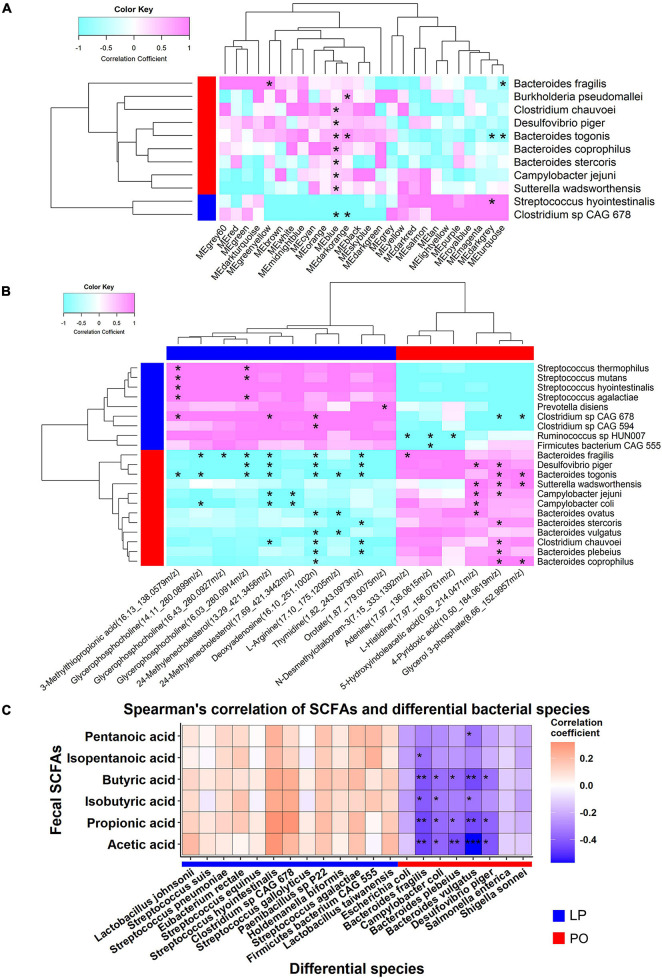
Correlations between metabolite features and differential bacterial species. **(A)** The correlations between differential bacterial species and metabolite modules that were clustered by WGCNA. **(B)** The correlations between differential bacterial species and differential metabolites. **(C)** The correlations between differential bacterial species and fecal SCFAs. Only those differential bacterial species which were detected the correlation are shown in the heatmap. The blue bar represents the bacterial species enriched at the LP stage; red bar indicates the bacterial species enriched at the PO stage. The stars indicate the significance threshold *P < 0.05, **P < 0.01, and ***P < 0.001 by Spearman’s rank correlation test.

A total of 26 metabolite modules were clustered from 7,857 metabolites using WGCNA according to the frequent intercorrelations between these metabolites (Supplementary Table 5). We first tested the Spearman’s rank correlations between these metabolite modules and the bacterial species showing differential enrichments between LP and PO stage in 39 samples with both metagenomic and metabolome data. Among these 26 modules, five modules showed significant correlations with 11 out of 56 differential bacterial species (Figure 5A and Supplementary Table 6). *Bacteroides togonis* was significantly associated with four metabolite modules, suggesting its important role in host metabolism. The MEblue module was associated with eight bacterial species including *Bacteroides togonis*, *Bacteroides coprophilus*, and *Bacteroides stercoris*. All these results demonstrated the relationships between serum metabolome and the gut microbiome.

We further analyzed the correlations between serum metabolite features and differential bacterial species. We focused on the 16 metabolite features (mz) from five metabolite modules showing correlations with differential bacteria and annotated to 12 KEGG pathways by online MetaboAnalyst 4.0. These 16 metabolites showed distinct enrichments between LP and PO stages (Wilcoxon rank-sum test, FDR < 0.05) and were significantly correlated with 21 out of 56 differential bacterial species (Figure 5B and Supplementary Table 7). 3-Methylthiopropionic acid (16.13_138.0579 m/z, cysteine and methionine metabolism) and glycerophosphocholine (16.03_280.0914 m/z, glycerophospholipid metabolism) were enriched at the LP stage and positively correlated with *Streptococcus* spp. (e.g., *Streptococcus thermophilus*, *Streptococcus mutans*, and *Streptococcus agalactiae*). Similarly, 24-methylenecholesterol (13.29_421.3456 m/z, steroid biosynthesis), deoxyadenosine (16.10_251.1002n, purine metabolism), and thymidine (1.82_243.0973 m/z, pyrimidine metabolism) were also enriched at the LP stage, and these metabolite features were negatively correlated with those bacterial species enriched at the PO stage, including *Bacteroides fragilis*, *Desulfovibrio piger*, *Bacteroides togonis*, and *Clostridium chauvoei*. The level of L-arginine was significantly higher at the LP stage, and showed significantly negative association with the abundances of *Bacteroides togonis*, *Bacteroides ovatus*, and *Bacteroides vulgatus* (Figure 5B). Additionally, 4-pyridoxic acid (10.50_184.0619 m/z, vitamin B6 metabolism) and glycerol 3-phosphate (8.66_152.9957 m/z, glycerolipid metabolism) were enriched at the PO stage, which were negatively associated with *Clostridium* sp. *CAG 678*, but showed positive correlations with most of the bacteria enriched at the PO stage (e.g., *Bacteroides togonis*, *Sutterella wadsworthensis*, and *Bacteroides coprophilus*). Moreover, 5-hydroxyindoleacetic acid (0.93_214.0471 m/z, Tryptophan metabolism) showed positive correlations with potential pathogens including *Desulfovibrio piger*, *Campylobacter jejuni*, and *Campylobacter coli* (Figure 5B). In general, all these results suggested the interaction between host metabolism and gut microbiome.

As for the relationships of fecal SCFA concentrations with differential bacterial species, we found that five bacteria enriched at the PO stage were negatively correlated with the concentrations of SCFAs (e.g., acetic acid, propionic acid, and butyric acid), including *Bacteroides fragilis*, *Bacteroides plebeius*, *Bacteroides vulgatus*, *Campylobacter coil*, and *Desulfovibrio piger* (Figure 5C). Particularly, *Bacteroides vulgatus* showed the most significant correlation with acetic acid (Spearman rank correlation, *r* = −0.57, *P* = 0.00013). All these results suggested that high abundances of these bacteria might inhibit the production of SCFAs in the gut at the PO stage.

## Discussion

The stage of parturition is crucially important for both sow and piglet. Dramatic changes of sow physiology status including hormones, metabolism, and gut microbiome should occur from late pregnancy to the postpartum period (Neuman and Koren, 2017; Huang et al., 2019). In the current study, we investigated the changes of the gut microbiome from LP to PO stage by combining both 16S rRNA gene and shotgun metagenomic sequencing analysis. The correlations between the changes of the gut microbiome, and the shifts of host serum metabolome and fecal SCFAs were evaluated. Through these analyses, our study provides more details about the changes of the gut microbiome, and serum and feces metabolome from LP to PO stage. The results also give a theoretical basis for prenatal-postnatal care of sows, and will benefit for the improvements of health and productivity of the sow herd by regulating gut microbiota.

Similar to the results that we reported previously (Huang et al., 2019), we revealed the alterations of the diversity and community structure of sow gut microbiome around parturition. A previous study reported that the abundances of *Proteobacteria* and *Actinobacteria* increased on average from the first to the third trimester of pregnancy (Koren et al., 2012). Similarly, in this study, we observed that the abundances of these two phyla continued to increase from LP to PO stage. Furthermore, we found that the relative abundances of *Bacteroides*, *Escherichia*, and *Desulfovibiro* increased from LP to PO stage, but the abundance of *Lactobacillus* was decreased. Many *Lactobacillus* strains were used as potential probiotics to maintain gut microbial homeostasis and promote intestinal health (Karczewski et al., 2010; Park et al., 2018). For instance, *Lactobacillus johnsonii* L531 can produce high levels of beneficial metabolites (e.g., butyric, acetic, and lactic acid) to treat *Salmonella* infection and reduce pathogens load in the intestines of piglets (He et al., 2019b). Indeed, we observed a moderate and positive correlation between *Lactobacillus* spp. (e.g., *Lactobacillus johnsonii* and *Lactobacillus taiwanensis*) and fecal SCFAs at the LP stage. Meanwhile, we identified a significant enrichment of the KEGG pathway about bacterial cell division and reproduction at the LP stage, which was positively correlated with *Lactobacillus johnsonii*, *Lactobacillus taiwanensis*, and *Paenibacillus* sp. *P22*. This agreed with a report that lactic acid-producing bacteria could promote microbial growth and bacteria propagation (Vandenbergh, 1993). Interestingly, previous studies found that *Lactobacillus* (e.g., *Lactobacillus reuteri*) can upregulate oxytocin release and systemic immune responses to achieve a wide array of health benefits involving in delivery, wound healing, metabolism, and energy balance, which are essential for normal labor and maternal health around parturition (Blanks, 2003; Erdman and Poutahidis, 2016). In contrast, many potential pathogens were abundant at the PO stage, including *Escherichia coli*, *Shigella sonnei*, *Salmonella enterica*, and *Campylobacter coli*, which have been reported to cause severe diseases such as diarrhea by invasion and inflammatory destruction of the colonic epithelium (Zhang et al., 2003; Sheppard and Maiden, 2015; Belotserkovsky and Sansonetti, 2018). Besides, *Bacteroides fragilis* and *Bacteroides vulgatus* are commensal organisms that can become opportunistic pathogens in certain individuals (Sears et al., 2008; Cuív et al., 2017). Moreover, these bacterial species were negatively correlated with the concentrations of fecal SCFAs, but positively associated with functional pathways about antimicrobial peptide resistance and sulfur metabolism, which might inhibit the growth of other symbiotic bacteria and influence microbiota composition after delivery (Cuív et al., 2017; Valguarnera and Wardenburg, 2019).

As we have known, SCFAs in the gut are generated by microbial fermentation and exhibit a wide range of physiological functions, such as anti-inflammation, immune regulation, and metabolism modulation (Ohira et al., 2017; Serino, 2019). SCFAs can increase host defense by enhancing the barrier integrity of the gut epithelium via histone deacetylase (HDAC) inhibition (Mathewson et al., 2016) and G-protein coupled receptors (GPCRs) activation (Macia et al., 2015). Additionally, SCFAs occasionally increase the systemic production of IgG and IgA to regulate pathogen-specific immune responses (Kim et al., 2016). The level of SCFAs was significantly lower at the PO stage, and their concentrations were negatively associated with the bacterial species that were enriched at the PO stage. This suggested that the protective effect of SCFAs on the intestinal epithelial barrier was reduced and the infection of pathogens may be further aggravated. Several potential pathogens (e.g., *Shigella sonnei* and *Salmonella enterica*) were indeed enriched at the PO stage after parturition. Some epidemiological evidences have shown that a detrimental living environment, the exposure of pathogens, and the impaired placental barrier can lead to the dysbiosis of gut microbiota and the flourish of pathogens during the perinatal period (Burton et al., 2016). However, *Bacteroides fragilis* and other *Bacteroides* strains can use glycan (e.g., exopolysaccharides) to produce propionate and acetate, and clean the potentially toxic structures by lysosome (Rios-Covian et al., 2016; Ko et al., 2017). This gave advantages to maintain the balance of gut microecology, which explained why sows did not show disease status although pathogens or opportunistic pathogens occurred at the PO stage.

We revealed that the changes of the gut microbiome from LP to PO stage were correlated with the alterations of host serum metabolome. The pathway of taurine and hypotaurine metabolism was enriched at the LP stage. Yu et al. (2016) reported that taurine played an important role in the regulation of gut microecology, and was beneficial to health through inhibiting pathogen growth and increasing the production of SCFAs. Additionally, we also found that the pathway related to arginine biosynthesis and metabolism was enriched by differential serum metabolites. The level of L-arginine was significantly higher at the LP stage and negatively associated with the abundances of *Bacteroides togonis*, *Bacteroides ovatus*, and *Bacteroides vulgatus* (Figure 5B and Supplementary Table 7). This result suggested that gut microbes may contribute to arginine metabolism around parturition. Previous studies have explored the possible connections between L-arginine and *Bacteroides* spp., and found the negative correlations between *Bacteroides ovatus* and arginine level (Ushijima and Seto, 1991; Sun et al., 2020). Previous studies confirmed that arginine supplementation was essential for maternal uterine and placental growth, embryonic and fetal survivals involving in nitric oxide and polyamines, and other functional amino acid syntheses during gestation, parturition, and lactation (Wu et al., 2013; Che et al., 2019). L-arginine can promote protein synthesis in the mammary glands of lactating sows and maintain small intestinal epithelial homeostasis through stimulating enterocyte migration in piglets (Rhoads et al., 2004; Mateo et al., 2007). Furthermore, 24-methylenecholesterol participating in steroid biosynthesis had a higher abundance at the LP stage. As we have known, significant changes in sex hormones would trigger the onset of parturition (Vannuccini et al., 2016). This metabolite was negatively correlated with the abundance of *Campylobacter jejuni* and *Campylobacter coli* at the PO stage, suggesting the roles of the gut microbiome in the changes of sex hormones around parturition.

Both the KEGG pathway related to the metabolism of cofactors and vitamins in the gut microbiome and the 4-pyridoxic acid from vitamin B6 metabolism in serum metabolome had a higher abundance at the PO stage. Vitamin B6 supplementation was regarded as necessary to prevent preterm birth and help the development of the fetal nervous system, as well as modulate steroid hormones during pregnancy and parturition (Allgood and Cidlowski, 1992; Salam et al., 2015). A previous report identified that vitamin B6 deficiency in diet would impair arginine biosynthesis and reduce propionate and butyrate levels of the cecum, which was derived by *Lachnospiraceae*_NK4A136 and *Bacteroides* (Mayengbam et al., 2020). Another report revealed that vitamin B6 can be produced by *B. fragilis* (Magnusdottir et al., 2015). In this study, the concentration of 4-pyridoxic acid was positively related to the relative abundance of *Bacteroides* spp. including *Bacteroides coprophilus*, *Bacteroides plebeius*, *Bacteroides stercoris*, and *Bacteroides togonis*. Besides, serum metabolite features associated with sphingolipid metabolism and glycerophospholipid metabolism were also enriched at the PO stage and showed positive correlations with *Bacteroides coprophilus* and *Bacteroides togonis*. Previous studies indicated that *Bacteroides* were involved in milk synthesis and nutrients supplementation for fetal development and growth (Walker and Iyengar, 2015).

In summary, we revealed the dynamic changes of sow gut microbiome, fecal SCFAs, and serum metabolome around parturition. The α-diversity of gut microbiota was decreased from LP to PO stage. We identified more than 30 genera and 56 bacterial species having significantly different abundances between LP and PO stages by metagenomic sequencing analysis. These differential species showed significant correlations with the changed serum and feces metabolites. A close correlation between the shifts of host serum metabolome and the changes of gut microbiome indicated the host-microbiota interactions during the perinatal period. However, the correlations of serum metabolites and gut microbiota were established only based on the association analysis, and the causality needs to be further confirmed by more experiments. The findings may provide new insights about the changes of sow physiological status around parturition from the aspects of the gut microbiome, fecal SCFAs and serum metabolome, and also provide the knowledge for guiding the maternal care of sows.

## Data Availability Statement

All 16S rRNA gene sequencing data and metagenomic sequencing data were submitted to the China National GeneBank database with the accession numbers CNP0001534 and CNP0000824.

## Ethics Statement

All animal procedures were conducted according to the guidelines for the care and use of experimental animals that were established by the Ministry of Agriculture and Rural Affairs (MARA) of China. The approval for pig husbandry and experiment was obtained from Animal Care and Use Committee (ACUC) in Jiangxi Agricultural University (No. JXAU2011-006).

## Author Contributions

LH designed the research and revised the manuscript. CC designed the research, wrote, and revised the manuscript. HF performed the experiments, analyzed the data, and wrote the manuscript. SK and ZC assisted in collecting samples. HJ, QL, and ML assisted in extracting the DNA from fecal samples. MH assisted in performing metabolomic experiments of LC-MS. JW and YZ assisted in quantitatively analyzing the concentrations of short-chain fatty acids. All authors read and approved the final manuscript.

## Conflict of Interest

The authors declare that the research was conducted in the absence of any commercial or financial relationships that could be construed as a potential conflict of interest.

## Publisher’s Note

All claims expressed in this article are solely those of the authors and do not necessarily represent those of their affiliated organizations, or those of the publisher, the editors and the reviewers. Any product that may be evaluated in this article, or claim that may be made by its manufacturer, is not guaranteed or endorsed by the publisher.

## Supplementary Material

Supplementary Figure 1 Effect of parities on the gut microbial composition. PCoA analysis did not detect the significant effect of parities on the gut microbial composition.

Supplementary Figure 2 The changes of gut microbial structures across four time points following the parturition. **(A)** Observed species (Sobs). **(B)** Chao index. **(C)** Shannon index. **(D)** Principal coordinate analysis (PCoA) showing the changes of gut microbial composition across four consecutive sampling times based on Bray-Curtis distances. Wilcoxon rank-sum test, ^∗^*P* < 0.05, ^∗∗^*P* < 0.01, and ^∗∗∗^*P* < 0.001.

Supplementary Figure 3 The bacterial taxa showing different enrichments between late pregnancy (LP) and postpartum (PO) stage. **(A)** The differential bacterial taxa at the phylum level. LDA score > 2. **(B)** The differential bacterial taxa at the genus level. LDA score > 2. **(C)** The differential bacterial taxa at the OTU level. LDA score > 3 (the differential bacterial taxa of 3 > LDA score > 2 showing in the Supplementary Table 2). LDA, linear discriminant analysis.

Supplementary Figure 4 Effect of parities on serum metabolomics profiles. PCoA analysis did not detect the significant effect of parities on serum metabolomics profiles.

Supplementary Table 1 Sample information and the experiments performed.

Supplementary Table 2 Bacterial taxa showing significant differences in their relative abundances between late pregnancy (LP) and postpartum (PO) stages with 16S rRNA gene sequencing data by LEfSe analysis. (LDA score > 2).

Supplementary Table 3 Bacterial species showing different enrichments between late pregnancy (LP) and postpartum (PO) stage with metagenomic sequencing data by LEfSe analysis. (LDA score > 2).

Supplementary Table 4 The KEGG pathways matched by differential metabolite features at late pregnancy (LP) and postpartum (PO) stage by online MetaboAnalyst 4.0.

Supplementary Table 5 Metabolite modules clustered by WGCNA according to the frequent intercorrelation between metabolites.

Supplementary Table 6 The correlations between differential bacterial species and metabolite modules.

Supplementary Table 7 The 16 differential metabolite features (mz) belonging to 5 metabolite modules and their associations with differential bacterial species.
