# Supplementary figures and images for: Deep Investigating the Changes of Gut Microbiome and Its Correlation With the Shifts of Host Serum Metabolome Around Parturition in Sows
Source: Front Microbiol. 2021 Sep 17;12:729039. doi: 10.3389/fmicb.2021.729039 (PMC8484970; doi:10.3389/fmicb.2021.729039)

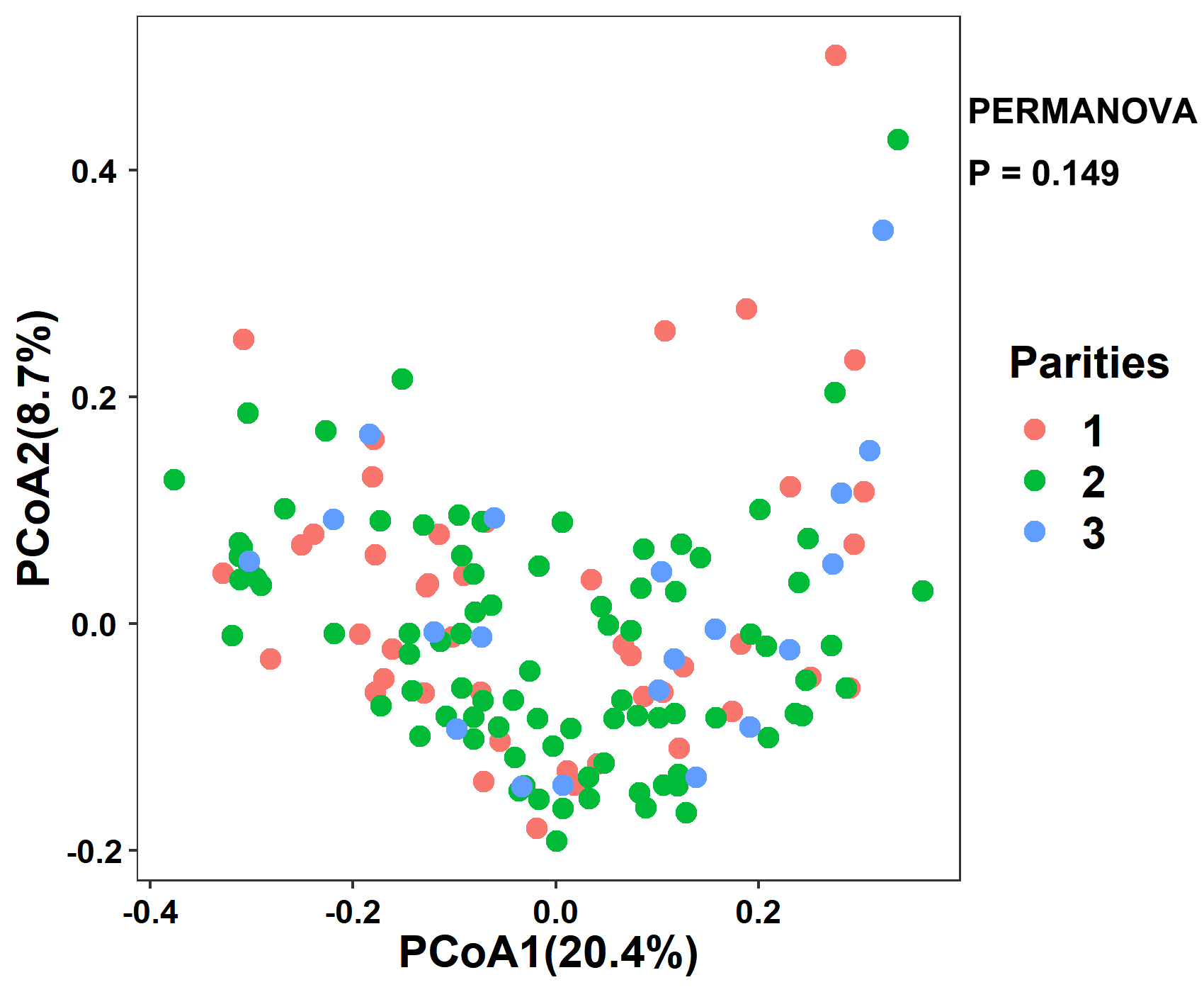

Supplement: Supplementary Figure 1 — Effect of parities on the gut microbial composition. PCoA analysis did not detect the significant effect of parities on the gut microbial composition. [file Image_1.TIFF]

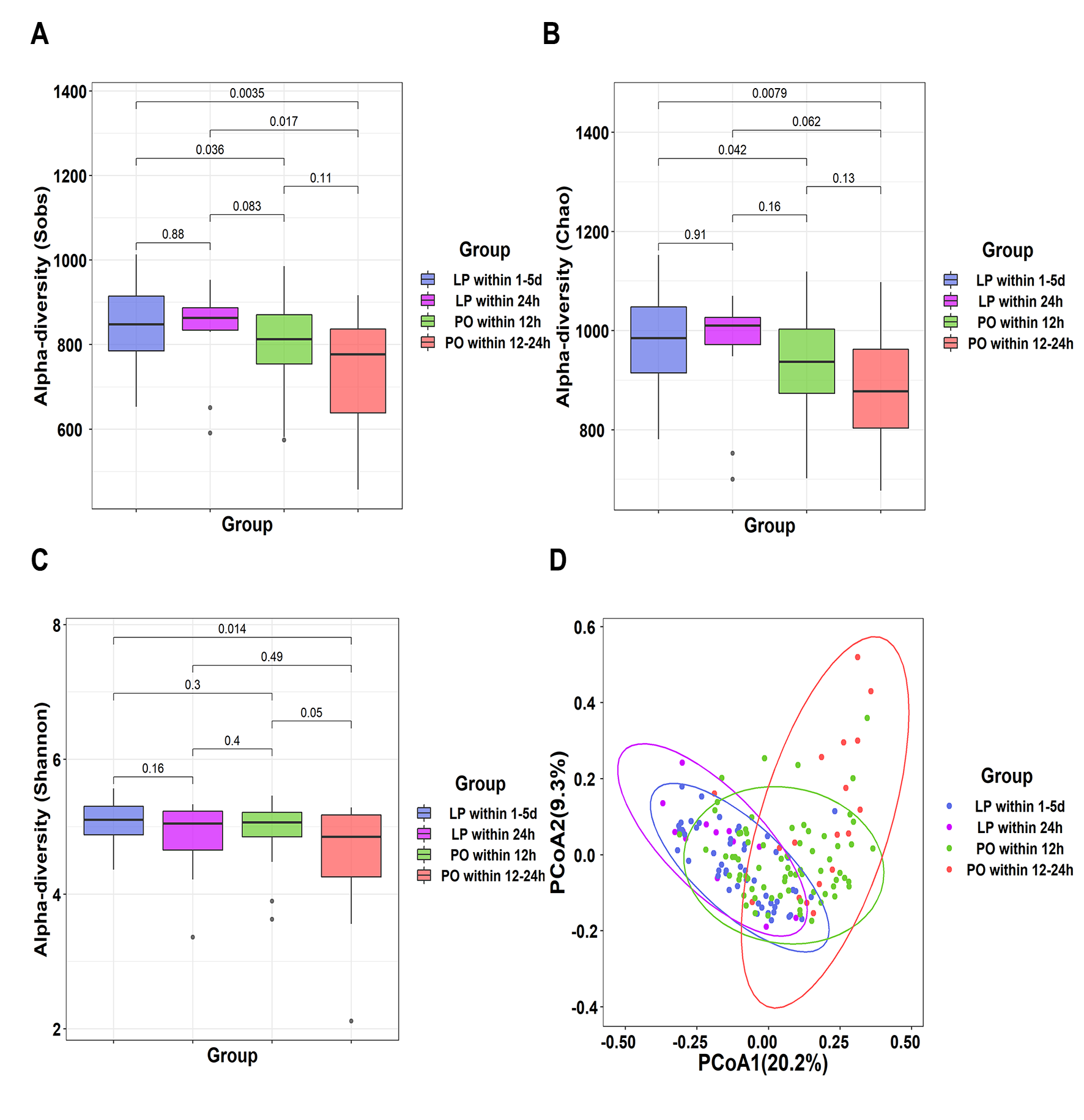

Supplement: Supplementary Figure 2 — The changes of gut microbial structures across four time points following the parturition. (A) Observed species (Sobs). (B) Chao index. (C) Shannon index. (D) Principal coordinate analysis (PCoA) showing the changes of gut microbial composition across four consecutive sampling times based on Bray-Curtis distances. Wilcoxon rank-sum test, ∗P < 0.05, ∗∗P < 0.01, and ∗∗∗P < 0.001. [file Image_2.TIF]

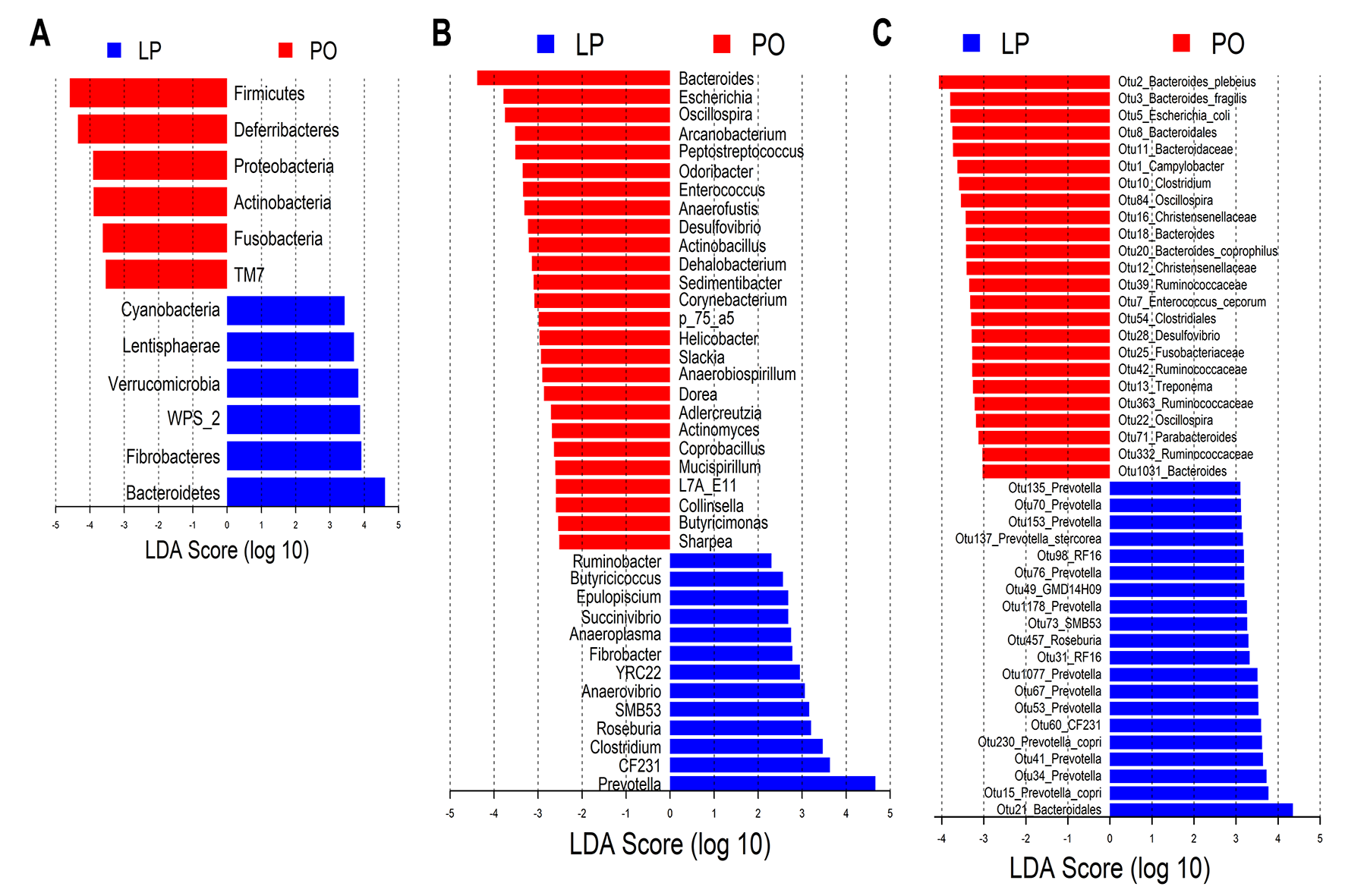

Supplement: Supplementary Figure 3 — The bacterial taxa showing different enrichments between late pregnancy (LP) and postpartum (PO) stage. (A) The differential bacterial taxa at the phylum level. LDA score > 2. (B) The differential bacterial taxa at the genus level. LDA score > 2. (C) The differential bacterial taxa at the OTU level. LDA score > 3 (the differential bacterial taxa of 3 > LDA score > 2 showing in the Supplementary Table 2). LDA, linear discriminant analysis. [file Image_3.TIF]

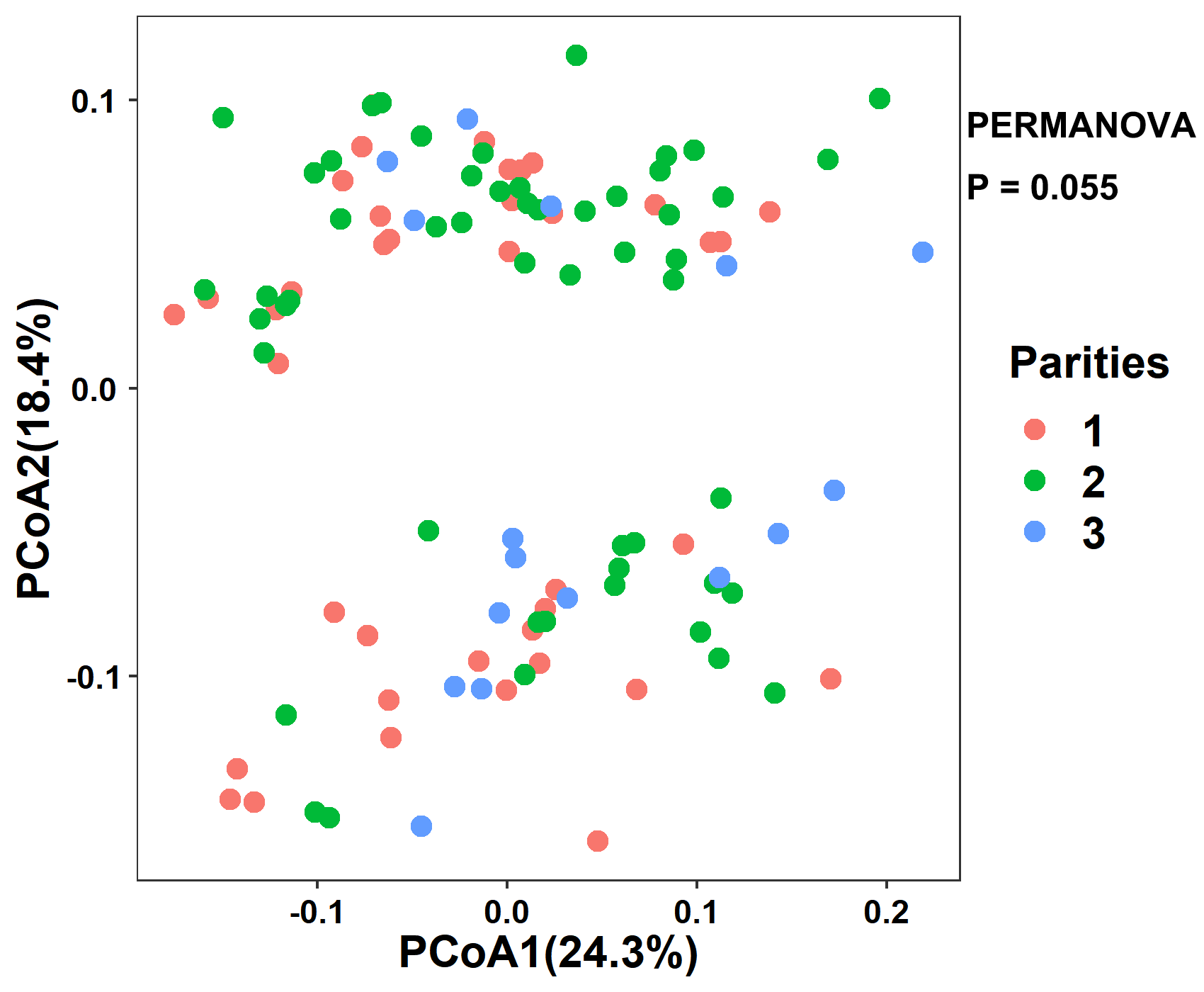

Supplement: Supplementary Figure 4 — Effect of parities on serum metabolomics profiles. PCoA analysis did not detect the significant effect of parities on serum metabolomics profiles. [file Image_4.TIFF]
